# Automatic Laser Glare Suppression in Electro-Optical Sensors

**DOI:** 10.3390/s150100792

**Published:** 2015-01-05

**Authors:** Gunnar Ritt, Bernd Eberle

**Affiliations:** Fraunhofer Institute of Optronics, System Technologies and Image Exploitation IOSB, Gutleuthausstr. 1, 76275 Ettlingen, Germany; E-Mail: bernd.eberle@iosb.fraunhofer.de

**Keywords:** laser dazzling, sensor protection, spatial light modulator, wavelength multiplexing

## Abstract

Progress in laser technology has led to very compact but nevertheless powerful laser sources. In the visible and near infrared spectral region, lasers of any wavelength can be purchased. Continuous wave laser sources pose an especially serious threat to the human eye and electro-optical sensors due to their high proliferation and easy availability. The manifold of available wavelengths cannot be covered by conventional safety measures like absorption or interference filters. We present a protection concept for electro-optical sensors to suppress dazzling in the visible spectral region. The key element of the concept is the use of a digital micromirror device (DMD) in combination with wavelength multiplexing. This approach allows selective spectral filtering in defined regions of interest in the scene. The system offers the possibility of automatic attenuation of dazzling laser radiation.

## 1. Introduction

Electro-optical sensors are widely used in many different applications, but they are susceptible to overexposure and to optical damage. A main source of threats to optical sensors are lasers. Since their development in 1960, the protection of human eyes and sensors against intended or unintended damage by laser radiation has been an ongoing research topic. Currently, this topic is receiving more attention due to the increasing misuse of low priced, compact and quite powerful laser sources. Compact laser sources with emission wavelengths in the entire visible and near-infrared spectral region and with output powers up to several watts are available. In civil environments, particularly aircraft crews as well as motorists are potential victims of dazzling attacks [1,2]. In such cases, the loss of vision can lead to fatal accidents. Besides the human eye, laser dazzling can also pose a severe problem to electro-optical sensors used in autonomous or surveillance systems [3], so adequate protection against dazzling is highly desirable.

Current laser protection measures are typically realized using conventional optical filters based on absorption or interference effects. Unfortunately, these filters work only for predefined wavelengths, but not beyond. Sophisticated protection concepts against dazzling and damaging are required, which work independent of the threatening wavelength and do not influence the system performance.

Numerous kinds of approaches were discussed in literature regarding realization concepts for laser protection [2]. Among them are active systems like shutters, frequency agile filters or spatial light modulators. In general, such active systems are useless against short-duration laser pulses since they suffer from the disadvantage of a finite response time. They also need a kind of laser warning sensor to detect the threatening laser light and a servo loop to react. Additionally, an electrical power supply is necessary. Nevertheless, active concepts are definitely attractive since the proliferation of compact continuous wave laser sources is rampant and uncontrollable.

Tomilin and Danilov, for example, described the use of spatial light modulators (SLM) as a protection measure against dazzling light sources [4]. The advantage of spatial light modulator technology is the possibility to build a protection measure, which can attenuate light coming from a specific direction within the field of view. At the same time, light coming from all other directions is not influenced. In order to realize such an operating principle, it is necessary to place the spatial light modulator in the intermediate focal plane of an optical system. Therefore, this method is primarily useful to protect electro-optical sensors against laser radiation.

With the implementation of *wavelength multiplexing*, we were able to significantly improve upon the concept of Tomilin and Danilov. This concept allows simultaneous spatial and spectral filtering of monochromatic light. In earlier work, we reported experimental setups based on liquid crystal SLM [5] and a digital micromirror device [6]. For these setups, complex algorithms were necessary to drive the system's control loop. In this article, we present an improved concept that enables a control loop realization without the need for high computing capacity. The new concept was realized as a compact demonstrator.

## 2. Operating Principle

*Wavelength multiplexing* is a technique, which Koester introduced to maintain the quality of images when transferred through optical fiber bundles [7]. The idea was to transmit the information from a single object point through a number of different fibers. It was realized by placing a double Amici prism (or direct vision prism) in front of the input optical system of a fiberscope, which imaged the object onto the entrance facet of the fiber bundle. Thus, the light from a given object point was spectrally divided and then transmitted through the fiber bundle. A corresponding dispersive element placed at the exit end of the fiber bundle reversed the dispersion. Therefore, image deterioration due to transmission loss of the fiber bundle was reduced, for example those caused by broken fibers or imperfect facet texture.

**Figure 1.**
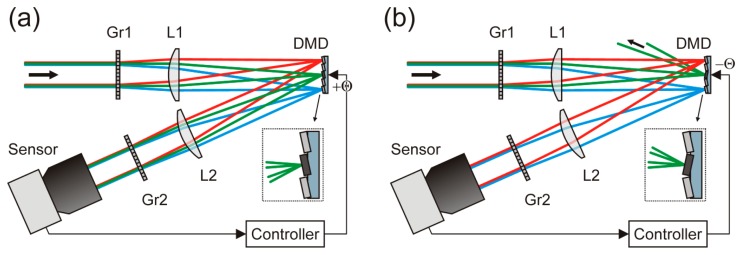
Scheme of a laser dazzling protection concept using a DMD. (**a**) Operation mode for regular imaging: all micromirrors are tilted towards the sensor. (**b**) Operation mode with high attenuation for dazzling light: the micromirrors which are exposed with dazzling laser light (here: the green rays) are tilted away from the sensor. Thus, the dazzling light will be strongly attenuated in the regular imaging path.

Figure 1 shows a schematic view of our protection concept combining a spatial light modulator with the technique of wavelength multiplexing. Light beams entering the optical setup are spectrally divided by a first dispersive optical element Gr1. A lens L1 focuses the beams onto the spatial light modulator. Here, we use a digital micromirror device (DMD) as spatial light modulator [8]. Since the DMD works in reflection, a folded beam path arises. A second lens L2, identical to lens L1, collimates the reflected light. A second dispersive optical element Gr2 reverses the dispersion of light by the first dispersive optical element Gr1. Finally, the light is imaged onto a camera sensor.

Usually, the optical setup would be operated in such a way that all light is directed towards the sensor by tilting all micromirrors to the +θ-state (see Figure 1a). If the sensor is dazzled by a laser (here: the green rays in the figure), the controller toggles just these micromirrors to the −θ-state which are exposed to the dazzling light (see Figure 1b). Thus, the dazzling light is reflected out of the beam path. The non-dazzling light coming from the same direction as the dazzling light, but with wavelengths different from the laser wavelength, will still be imaged onto the camera sensor (here: the red and blue rays in the figure). Such a setup suppresses only the threatening laser light, while not affecting the remaining safe radiation from the scene.

## 3. Optical Setup

**Figure 2.**
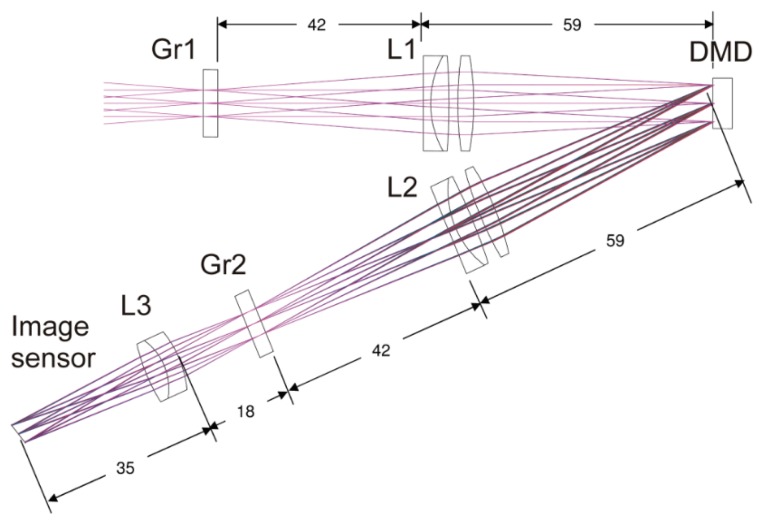
Optical layout of the protection setup. A DMD is located in the intermediate focal plane of a Keplerian telescope (achromatic lenses L1 and L2). Wavelength multiplexing is implemented by the use of two diffraction gratings (grating Gr1 and Gr2). In contrast to the scheme of Figure 1, the gratings are aligned so that the spectral dispersion takes place in a plane perpendicular to the plane of reflection at the DMD. The distance values are given in millimeters.

Figure 2 shows the optical layout of the latest experimental setup. Blazed gratings (Gr1 and Gr2, 300 grooves/mm) are used to implement the wavelength multiplexing. The use of gratings instead of prisms (in contrast to the prisms used in the original setup of Koester [7]) allows one to build a more compact setup on the cost of light transmittance. The gratings offer a diffraction efficiency of more than 60% for wavelengths in the range from 450 nm to 700 nm. The absolute efficiency is defined by the ratio of the power diffracted into the preferred order to the total incident power. The gratings were aligned so that the spectral wavelength separation takes place in a plane perpendicular to the plane of reflection at the DMD. Therefore, the spectral dispersion of the wavelengths is not visible in the optical layout.

The DMD is placed at the position of the intermediate focal plane of the telescope formed by two achromatic lenses, each with a focal length of 50 mm (achromatic lens L1 and L2). The light is reflected by the DMD with an angle of 24° to the normal of the DMD surface. A third lens (achromatic lens L3) with a focal length of 32 mm forms the final image. The focal plane of the achromatic lens L2 and the plane of the DMD do not coincide. Therefore, the image plane is tilted with regard to the optical axis. In our setup, we used a color CMOS sensor VRmMS-12/C from VRmagic GmbH (Mannheim, Germany), which is aligned according to the Scheimpflug principle.

We use a 0.7″ XGA DMD from Texas Instruments (Dallas, TX, USA) as spatial light modulator. This DMD offers 1024 × 768 micromirrors with a pitch of 13.68 μm. Each mirror can be toggled from a +12° to a −12° state; the axis of rotation is oriented at an angle of 45° with respect to the edges of the array. The DMD efficiency for wavelengths between 420 nm and 700 nm is specified to be 68%, which includes the transmission of the protection window (∼97%), the fill factor (∼92.5%), the mirror reflectivity (∼88%) and the diffraction efficiency (∼86%). This value is defined as the amount of light that is reflected specularly by the array. Table 1 lists the specifications of the complete system.

## 4. Control Loop Implementation

The task of the controller indicated in Figure 1 is to detect laser dazzling and to drive the DMD automatically according to the actual situation. In case of incoming dazzling laser light, the controller has to toggle the appropriate pixels of the SLM in order to suppress the overexposure. The activation of the correct pixels depends: (1) on the direction from where dazzling originates and (2) on the wavelength of the dazzling laser.

The direction from where the dazzling originates can be figured out directly from the camera image. However, the wavelength of the dazzling laser is a priori not known. This means that the exact location where the dazzling light is focused onto the SLM is not known. In the first instance, only information on the appropriate columns of pixels is given since the dispersion of light takes place in a vertical direction due to the orientation of the grating.

For the case of a color camera to be protected, the information on the dazzling wavelength can be deduced directly from the color information contained in the camera's images. The overexposed regions on the detector are surrounded by unimpaired pixels, which contain the necessary information about the searched wavelength. For instance, in a situation as shown in Figure 4, an observer could immediately say that some red laser light affects the sensor. A good estimation of the dazzling wavelength can be derived by an analysis of the color values of the non-saturated contour pixels surrounding the overexposed area. A detailed description of this wavelength estimation algorithm is given in an earlier publication [6].

**Figure 3.**
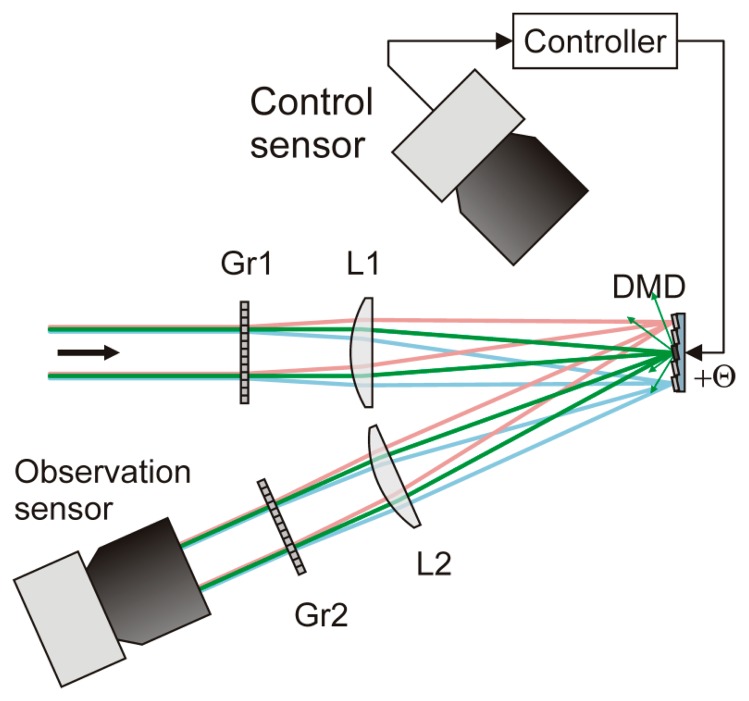
Scheme of the optical setup for an improved implementation of the control loop. A specific control sensor detects stray light occurring at the DMD due to intensive laser radiation. Thus, the controller can activate the corresponding micromirrors without the need for a wavelength analysis.

For this approach, the maximum frame rate of the sensor is not limited by the hardware but rather by the processing time for the wavelength estimation. A specific value for the processing time cannot be stated since the computational process depends on the complexity of the scene. For example, camera images with larger dazzled areas require more processing time than images with small dazzled areas. Multiple dazzling laser sources in the scene also result in a higher processing time since the wavelength estimation algorithm has to be utilized separately for each overexposed area in the camera image. To avoid restrictions to the sensor frame rate due to the software, an improved optical setup for an easier implementation of the control loop was realized. In Figure 3, a scheme of the improved optical setup is shown. A specific “control sensor” was integrated, which directly observes the DMD. In case of laser dazzling, the laser radiation focused onto the DMD induces intense stray light from the edges of the micromirrors and the substrate underneath. The (monochrome) control sensor directly monitors the centers of stray light and enables the controller to compute a corresponding pixel pattern for the DMD. The computation consists of a simple thresholding operation on the control sensor image and a subsequent image transformation (homography).

This approach to realize the control loop has no need for knowledge about the laser wavelength. Furthermore, high intensity radiation by broadband light sources (e.g., the sun) induces only weak stray light signals since broadband radiation is dispersed over a larger area of the DMD.

## 5. Results

### 5.1. Field Trials

**Figure 4.**
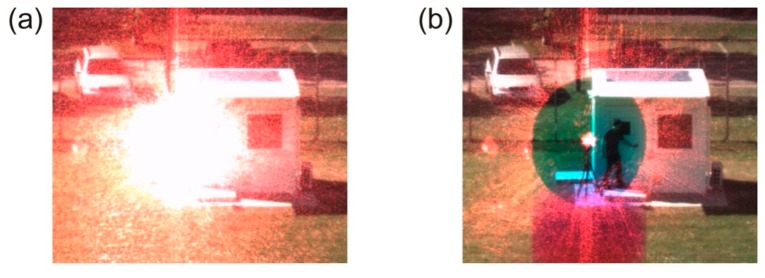
(**a**) Disturbed scene: Laser radiation dazzles the camera. Filtering of dazzling laser light is deactivated. (**b**) View of the scene when the filtering of dazzling laser light is activated. As a side effect, a vertically arranged color distortion is recognizable, but the geometrical details are visible.

The results of a field trial are shown in Figure 4. A continuous wave laser (wavelength: 660 nm, output power: 2.7 mW) was placed at a distance of 73 m to the sensor. These parameters would correspond to a laser output power of 81 mW at a distance of 400 m. When the laser was switched on, a large part of the central field of view was completely dazzled (see Figure 4a). As soon as the control loop of the system was activated, the dazzling laser radiation was strongly attenuated (see Figure 4b). Since a band of wavelengths was extracted out of the imaging path, a color distortion occurred, but the details in close vicinity to the laser (e.g., the person) are visible.

### 5.2. Laboratory Tests

In laboratory tests, the maximum attenuation of monochromatic light and the system transmittance were measured as a function of the wavelength of incident radiation. For the measurements, a coherent white light source (Koheras SuperK Extreme, Birkerød, Denmark) equipped with an acousto-optic tunable filter was used to produce narrow-band radiation adjustable in the visible spectral range.

**Figure 5.**
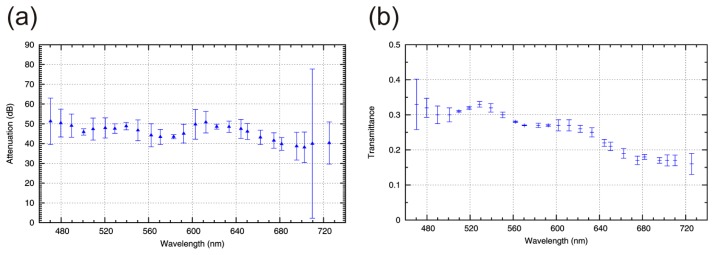
(**a**) Measured attenuation of monochromatic radiation as a function of wavelength. (**b**) System transmittance as a function of wavelength.

For the measurement of the maximum attenuation, a set of calibrated neutral density filters was used to adjust the radiation power. First, the signal of the observation sensor was registered with the DMD in the “bright state”. Subsequently, the signal of the observation sensor was measured with the DMD in the “dark state”. For both measurements, the power of radiation was adjusted by removing neutral density filters in such a way that the sensor signal was around 85% of the sensor's dynamic range. By calculating the ratio of both measured values in consideration of the neutral density filters, the maximum attenuation was calculated. Figure 5a shows the measured attenuation of monochromatic light as a function of the wavelength. The mean attenuation in the visible spectral region (470 nm–725 nm) is 45.5 dB.

To estimate the system transmittance, the radiation power measured with a photodiode power sensor (Ophir PD300-1W, Jerusalem, Israel) was compared in front of entrance optics and in front of the observation sensors with the DMD in the “bright state”. In Figure 5b the wavelength dependent transmittance of the system is plotted.

Both curves show a similar cyclic variation with wavelength. This variation can be explained by diffraction of light at the DMD due to the grid-like arrangement of the micromirrors as is explained in more details in Section 5.3. A mean transmittance of 0.26 was measured within the aforementioned spectral range.

### 5.3. Theoretical Estimation of the Maximum Attainable Attenuation

The measured curve for the attenuation of monochromatic light in Figure 5a exhibits a cyclic variation. The period of this variation increases with wavelength. Since a DMD represents a blazed grating, this behavior can be modeled by diffraction theory. Diffraction of light at the DMD also implies that an incident laser beam can never be removed completely out of the beam path by tilting all micromirrors to the dark state. There will always be light diffracted to lens L2 (see Figure 1), although these diffraction orders might be of very low intensity.

**Figure 6.**
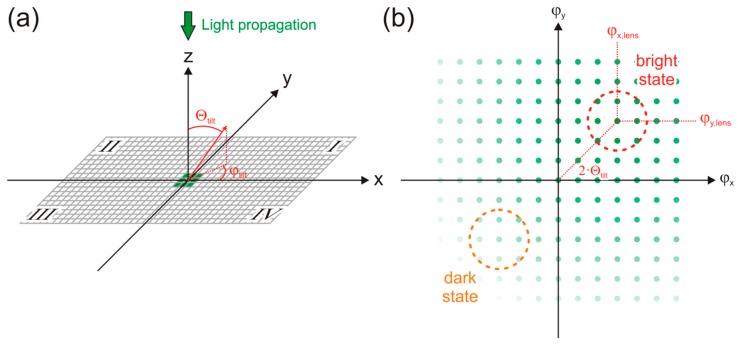
Diffraction of monochromatic light at the DMD. (**a**) The DMD shall be located in the x/y plane of a Cartesian coordinate system. Light propagates along the *z*-axis and illuminates a small area of the DMD (here: 3 × 3 micromirrors, marked in green). The red vector gives the tilting of the micromirrors. (**b**) Scheme of the diffraction pattern in angle space. The diffraction orders gathered by lens L2 (see Figure 1) in the “bright state” and in the “dark state” are marked by red and orange areas, respectively.

For the calculations, we assume that the DMD is placed at the x/y-plane of a Cartesian coordinate system, whereas the longer edge of the DMD is aligned parallel to the *x*-axis. A laser beam propagates along the *z*-axis. This geometry is depicted in Figure 6a. The micromirrors are tilted by an angle Θ_tilt_ with respect to the *z*-axis towards the direction of the diagonal of quadrant I of the x/y-plane (*φ_tilt_* = 45°). Due to the diffraction of the laser light, a two dimensional diffraction pattern is obtained, as shown in Figure 6b. Since the DMD represents a blazed grating, the distribution of intensity into the single diffraction orders is different. Diffraction orders in the angular area of 2Θ_tilt_ are preferred.

The exact intensity distribution is given by the superposition of the diffraction pattern of a grid and the diffraction pattern of a quadratic aperture (here: a micromirror) [9]:

(1) $$I\left(\varphi_{x},\varphi_{y}\right)=I_{\text{grating}}\left(\varphi_{x},\varphi_{y}\right)\cdot I_{\text{aperture}}\left(\varphi_{x},\varphi_{y}\right)$$

The two-dimensional diffraction pattern of the grating is given by:

(2) $$I_{\text{grating}}\left(\varphi_{x},\varphi_{y}\right)∼{\left(\frac{\sin\left(N\pi \frac{g}{\lambda }\cdot \sin\varphi_{x}\right)}{\sin\left(\pi \frac{g}{\lambda }\cdot \sin\varphi_{x}\right)}\right)}^{2}\cdot {\left(\frac{\sin\left(N\pi \frac{g}{\lambda }\cdot \sin\varphi_{y}\right)}{\sin\left(\pi \frac{g}{\lambda }\cdot \sin\varphi_{y}\right)}\right)}^{2},$$

where *N* is the amount of illuminated micromirrors, *g* the grating constant and λ the wavelength of the laser.

The diffraction pattern of a quadratic aperture is given by:

(3) $$I_{\text{aperture}}\left(\varphi_{x},\varphi_{y}\right)∼{\text{sinc}}^{2}\left(\pi \frac{b}{\lambda }\cdot \left(\sin\varphi_{x}-\sin\varphi_{x,0}\right)\right)\cdot {\text{sinc}}^{2}\left(\pi \frac{b}{\lambda }\cdot \left(\sin\varphi_{y}-\sin\varphi_{y,0}\right)\right).$$

Here, *b* is the edge length and the angles *φ_x,0_* and *φ_y,0_* describe the center of the diffraction pattern, which does not coincide with the origin of the coordinate system (blazed grating).

To estimate the maximum attainable attenuation, the calculated intensity distribution has to be integrated within the angular area that is covered by lens L2. This has to be conducted for both the bright state and the dark state of the DMD. The ratio of both integration values gives the attainable attenuation.

The integration area for the bright state of the DMD is marked in Figure 6b by a red circle. For the dark state of the DMD, the angular area marked in orange was used for the calculations. This area is given by mirroring the red area at the origin. For the calculations, the following system parameters were taken as a basis:

- Grating constant (grating pitch): *g* = 13.68 μm
- Fill factor: *f* = 92.5%
- Micromirror tilt angle: Θ_tilt_ = 12, *φ_tilt_* = 45
- Number of illuminated micromirrors: 3×3 (equivalent to a laser spot size of 40 μm)
- Pixel edge length (size of the active micromirror area): $b=\sqrt{f\cdot g^{2}\cdot \cos\Theta_{\text{tilt}}}=13.01\;\mu \text{m}$
- Lens: *f* = 50 mm, *D* = 18 mm, Θ_lens_ = 24, *φ_lens_* = 45 ⇒ *φ_x,lens_* = *φ_y,lens_* ≈17.5

**Figure 7.**
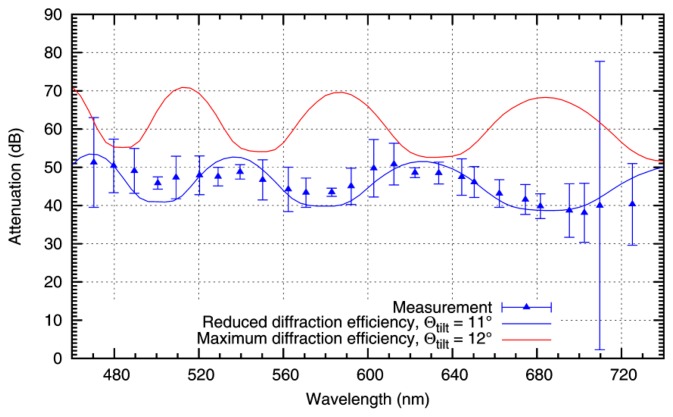
Comparison of the measured attenuation of monochromatic light with theoretical calculations. For ideal conditions (red curve), the maximum attainable attenuation is considerably higher than the measured values. The blue curve represents a calculation for fitted system parameters and is in good agreement with the measurement.

For these values, the center of the single-slit diffraction pattern coincides with the center of the area gathered by the lens. The result of the calculations for different wavelengths is plotted in Figure 7 as a red curve, together with the measuring values from Figure 5a.

It is apparent that the theoretically attainable attenuation is higher than the measured values (theoretical mean attenuation of 60.2 dB, to be compared with 45.5 dB measured). Additionally, it is obvious that the cyclic variation of the theoretical curve does not fit the behavior of the measuring values.

The lower measured attenuation can be explained by the non-ideal diffraction behavior of the DMD. The aluminium coating of the micromirrors exhibits a surface roughness and a non-ideal reflectivity. Furthermore, stray light occurs at the edges of the micromirrors and from the substrate, reducing the theoretical value.

The positions of the maxima and minima of the theoretical curve arise from the blaze angle Θ_tilt_. The DMD is specified with a typical tilt angle of 12° for the micromirrors, and minimum and maximum values between 11° and 13°, respectively. For the device in use, a deviation from the typical value of 12 is probable.

In Figure 7, an additional theoretical curve is plotted in blue, where a tilt angle Θ_tilt_ = 11 was assumed. The position of L2 was left unchanged. Additionally, the mean attenuation was adapted with a factor of 0.75. This value approximates diffraction efficiencies, which are achieved with real diffraction gratings. The resulting curve is in good agreement with the measured values.

## 6. Conclusions/Outlook

We presented a novel concept to protect electro-optical sensors from dazzling by continuous wave laser sources. It is based on a digital micromirror device (DMD) combined with wavelength multiplexing. This concept allows for selective spectral filtering within arbitrary areas of the sensor's field of view. Thus, all visual scene details will be preserved and not blacked out as in simple concepts without wavelength multiplexing.

A simple but effective control loop system to drive the DMD was realized. Main part of the control loop system is a specific detector, which observes stray light at the DMD at the incidence of intense monochromatic light radiation. With this additional component in place, the necessary pixel pattern to counter-affect dazzling can be computed by a simple image transformation (homography). Knowledge about the wavelength of the threatening laser is not needed, and the computing speed is dramatically increased.

## Figures and Tables

**Table 1.** Specifications of the complete system.

Effective focal length	32 mm
Entrance pupil diameter	5 mm
Field of view	8.0 (h) × 5.2° (v)
Spectral range	400 nm–700 nm
RMS spotsize	<20 μm
Vignetting	<5%
System transmittance (see Section 5.2)	26%
Mean attenuation of laser light (see Section 5.2)	45.5 dB
